# Altered Fermentation Performances, Growth, and Metabolic Footprints Reveal Competition for Nutrients between Yeast Species Inoculated in Synthetic Grape Juice-Like Medium

**DOI:** 10.3389/fmicb.2018.00196

**Published:** 2018-02-13

**Authors:** Stephanie Rollero, Audrey Bloem, Anne Ortiz-Julien, Carole Camarasa, Benoit Divol

**Affiliations:** ^1^Department of Viticulture and Oenology, Institute of Wine Biotechnology, Stellenbosch University, Stellenbosch, South Africa; ^2^UMR SPO, INRA, SupAgroM, Université de Montpellier, Montpellier, France; ^3^Lallemand SAS, Blagnac, France

**Keywords:** *S. cerevisiae*, non-*Saccharomyces* yeasts, yeast interactions, nutrient competition, fermentative aromas, wine

## Abstract

The sequential inoculation of non-*Saccharomyces* yeasts and *Saccharomyces cerevisiae* in grape juice is becoming an increasingly popular practice to diversify wine styles and/or to obtain more complex wines with a peculiar microbial footprint. One of the main interactions is competition for nutrients, especially nitrogen sources, that directly impacts not only fermentation performance but also the production of aroma compounds. In order to better understand the interactions taking place between non-Saccharomyces yeasts and S. cerevisiae during alcoholic fermentation, sequential inoculations of three yeast species (*Pichia burtonii, Kluyveromyces marxianus, Zygoascus meyerae*) with *S. cerevisiae* were performed individually in a synthetic medium. Different species-dependent interactions were evidenced. Indeed, the three sequential inoculations resulted in three different behaviors in terms of growth. *P. burtonii* and *Z. meyerae* declined after the inoculation of *S. cerevisiae* which promptly outcompeted the other two species. However, while the presence of *P. burtonii* did not impact the fermentation kinetics of *S. cerevisiae*, that of *Z. meyerae* rendered the overall kinetics very slow and with no clear exponential phase. *K. marxianus* and *S. cerevisiae* both declined and became undetectable before fermentation completion. The results also demonstrated that yeasts differed in their preference for nitrogen sources. Unlike *Z. meyerae* and *P. burtonii, K. marxianus* appeared to be a competitor for *S. cerevisiae* (as evidenced by the uptake of ammonium and amino acids), thereby explaining the resulting stuck fermentation. Nevertheless, the results suggested that competition for other nutrients (probably vitamins) occurred during the sequential inoculation of *Z. meyerae* with *S. cerevisiae*. The metabolic footprint of the non-*Saccharomyces* yeasts determined after 48 h of fermentation remained until the end of fermentation and combined with that of *S. cerevisiae*. For instance, fermentations performed with *K. marxianus* were characterized by the formation of phenylethanol and phenylethyl acetate, while those performed with *P. burtonii* or *Z. meyerae* displayed higher production of isoamyl alcohol and ethyl esters. When considering sequential inoculation of yeasts, the nutritional requirements of the yeasts used should be carefully considered and adjusted accordingly. Finally, our chemical data suggests that the organoleptic properties of the wine are altered in a species specific manner.

## Introduction

Spontaneous alcoholic fermentation is a complex microbial process that involves diverse yeast species. These yeast species are mostly characterized by large and predominant populations of non-*Saccharomyces* species in grape juice and at early stages of fermentation. Thereafter, *Saccharomyces cerevisiae* dominates and completes the fermentation (Fleet, 1993, 2003). Until recently, non-*Saccharomyces* have been associated with spontaneous and unpredictable fermentation which may lead to stuck or sluggish fermentations. However, some of these species have now garnered interest in winemaking practices because of their positive impact on the wine quality and complexity (Ciani et al., 2006; Fleet, 2008; Anfang et al., 2009; Viana et al., 2009, 2011; Andorrà et al., 2010; Jolly et al., 2014) and in an attempt to reach new consumer's markets.

As the majority of the non-*Saccharomyces* yeasts found in grape juice are unable to ferment to dryness, the use of controlled mixed or sequential fermentations of non-*Saccharomyces* yeasts together with *S. cerevisiae* appears to be an appropriate process to combine a diversification of the wine styles and a reliable and complete fermentation (Romano et al., 1997; Sadoudi et al., 2012; Gobbi et al., 2013). Although a massive amount of cells of *S. cerevisiae* is typically used for inoculation, many studies have shown that indigenous or commercial non-*Saccharomyces* strains are not completely suppressed, and may persist during other fermentative stages (Ciani et al., 2010; Medina et al., 2012; Lopez et al., 2014; Wang et al., 2015).

The main concern about the use of mixed/sequential cultures of different yeasts is the probable occurrence of complex interactions between the organisms (Fleet, 2003; Alexandre et al., 2004; Barbosa et al., 2015). These interactions can have a desirable or a detrimental effect on the fermentation process and the organoleptic properties of wines. The main positive influence of the mixed/sequential inoculation of non-*Saccharomyces* yeasts with *S. cerevisiae* is the increase in the concentration of desirable compounds, such as esters (Moreira et al., 2005, 2008; Viana et al., 2009; Renault et al., 2015). In 2006, Howell et al. showed different profiles of compounds in wines obtained by co-culture fermentation from those made in mono-culture (Howell et al., 2006). These authors also demonstrated that the combination of volatile aromas found in mixed cultures of *Saccharomyces* yeasts was distinctly different from that obtained by blending together mono-culture wines indicating a clear metabolic interaction between the yeasts. Nevertheless, the initial rapid growth of some non-*Saccharomyces* strains may have a negative impact on the metabolism and physiology of *S. cerevisiae* leading to sluggish or stuck fermentations. Competition for nutrients seemed to be one of the main causes for incomplete fermentations in non-*Saccharomyces*/*Saccharomyces* co-cultures. A more complete understanding of nutrient requirements for the non-*Saccharomyces* yeasts is necessary to better conduct the mixed and sequential fermentations in terms of nutrition to avoid sluggish or stuck fermentations. The impact of nutrient limitation on mixed/sequential cultures wine fermentation has been poorly studied. However, deficiency in nitrogen and some vitamins such as thiamine and pantothenic acid has been associated with sluggish wine fermentations performed with *S. cerevisiae* (Bataillon et al., 1996; Bisson, 1999; Blateyron and Sablayrolles, 2001; Wang et al., 2003; Bohlscheid et al., 2007). Medina et al. (2012) were among the first authors to highlight competition for nitrogen between *S. cerevisiae* and *Hanseniaspora vinae* or *Metschnikowia pulcherrima*, especially when the initial nitrogen content was too low. Nevertheless, in the latter study, only the total yeast assimilable nitrogen (YAN) was monitored and not the individual consumption of each nitrogen source by non-*Saccharomyces* yeasts. In 2014, Taillandier et al. reported a similar result when *Torulaspora delbrueckii* was inoculated together with *S. cerevisiae*. The presence of *H. guilliermondii* had a strong influence on the gene expression of *S. cerevisiae*, in particular on genes involved in the biosynthesis of vitamins as well as uptake and biosynthesis of amino acids (Barbosa et al., 2015). These results underlined the importance of competition for nitrogen and vitamins between yeast species. Kemsawasd et al. (2015b) showed that certain nitrogen sources were beneficial for all yeast species while others were only beneficial to specific species. Overall, the influence of nitrogen sources on yeast growth and fermentation performance differed between species, with *T. delbrueckii* and *H. uvarum* being the most similar to *S. cerevisiae*. Recently, Gobert et al. (2017) determined the order of uptake of nitrogen sources of three non-*Saccharomyces* yeast strains (*M. pulcherrima, Starmerella bacillaris*, and *Pichia membranifaciens*) inoculated as pure cultures in grape juice. Species-dependent differences were evidenced, but these did not impact *S. cerevisiae*'s fermentation and growth performances in sequential cultures. However, the consumption of different concentrations of nitrogen sources by the non-*Saccharomyces* yeasts impacted the organoleptic properties of the final wines.

Rollero et al. (submitted) have recently determined the preferences in terms of nitrogen sources for 10 non-conventional wine yeasts isolated from South African grape juices. This work highlighted some differences with *S. cerevisiae* as the consumption of GABA or few amount of ammonium (as *Zygoascus meyerae* or *Pichia burtonii*) but some strains, *Kluyveromyces marxianus* for instance, displayed the same preferences than *S. cerevisiae*. Specific aroma profiles for these strains were also identified in pure culture and could be interesting for the organoleptic properties of wines. In summary, the few studies published in literature suggest that, when non-*Saccharomyces* yeasts are co-inoculated with *S. cerevisiae*, competition for nutrients occurs and may have dire impact on fermentation.

The aim of our study was to evaluate the effect of nitrogenous nutrient consumption in a synthetic fermentation broth by three non-*Saccharomyces* strains (*P. burtonii, Z. meyerae*, and *K. marxianus*) selected during a previous study (Rollero et al. submitted) during sequential inoculation with *S. cerevisiae* on their growth, fermentation performances, and aroma production. Possible interactions and competitions for nutrients, in particular nitrogen sources, between *S. cerevisiae* and selected non-*Saccharomyces* yeasts were also assessed in order to optimize sequential fermentations, to manage nutrient supplementation adequately and ultimately prevent stuck or sluggish fermentations.

## Materials and methods

### Yeasts strains and preculture conditions

The fermentations were performed with the commercial wine strain *Saccharomyces cerevisiae* Lalvin EC1118® (Lallemand SA, Montreal, Canada) and three non-*Saccharomyces* yeasts isolated from South African grape juices (IWBT collection, Stellenbosch, South Africa), namely *Kluyveromyces marxianus* IWBT Y885, *Zygoascus meyerae* IWBT Y826, and *Pichia burtonii* IWBT Y951. The cryopreserved yeast cultures were thawed at room temperature and streaked on Yeast Peptone Dextrose (YPD) agar (Biolab-Merck, Modderfontein, South Africa). Starter cultures of all yeast strains were prepared by inoculating a single colony into 5 ml YPD broth for each strain. The cultures were incubated at 30°C on a test tube rotating wheel for 24 h. These starter cultures were used to inoculate YPD precultures at an initial cell density of 1 × 10^6^ cells/ml which were incubated at 30°C with shaking (125 rpm) for 9 h. In an attempt to deplete the reserves of nitrogen sources present in the cells, the yeasts were incubated for 4 h (*P. burtonii*), 6 h (*K. marxianus*), or 8 h (*Z. meyerae* and *S. cerevisiae*) in YNB containing neither amino acid nor ammonium (Difco Laboratories) supplemented with 20 g/l of glucose at 30°C with shaking (125 rpm). The growth in this medium was monitoring every 2 h until the end of growth corresponding to the depletion in nitrogen.

Sequential mixed cultures were performed with the inoculation of one of the non-*Saccharomyces* yeasts 48 h before *S. cerevisiae* yeast. A pure culture with only *S. cerevisiae* was also carried out. All the strains were inoculated from the preculture at 1 × 10^6^ cells/ml.

### Fermentations conditions and sampling

Fermentations were carried out in synthetic medium (SM) that simulates standard grape juice (Bely et al., 1990). The SM used in this study contained 230 g/l of sugar (115 g/l of glucose and 115 g/l of fructose); 2.5 g/l of potassium L-tartrate; 3 g/l of malic acid; 0.2 g/l of citric acid; 1.14 g/l of potassium hydrogen phosphate; 0.44 g/l of magnesium sulfate heptahydrate; 1.23 g/l of calcium chloride dehydrate; vitamins (mg/l): myo-inositol (100), calcium pantothenate (1), thiamin hydrochloride (0.5), nicotinic acid (2), pyridoxine hydrochloride (2), biotin (0.125), PABA.K (0.2), riboflavin (0.2), folic acid (0.2); trace elements (μg/l): manganese (II) chloride tetrahydrate (200), zinc chloride (135), iron chloride (30), copper chloride (15), boric acid (5), cobalt nitrate hexahydrate (1), sodium molybdate dehydrate (25), potassium iodate (10).

The nitrogen sources comprised ammonium chloride and amino acids. The composition of the stock solution of amino acids and ammonium was (in g/l): tyrosine (1.8), tryptophan (17.9), isoleucine (3.2), aspartate (4.4), glutamate (12.0), arginine (37.4), leucine (4.8), threonine (7.5), glycine (1.8), asparagine (5.3), glutamine (50.5), alanine (14.5), valine (4.4), methionine (3.1), phenylalanine (3.7), serine (7.8), histidine (3.2), lysine (1.7), GABA (14), cysteine (1.3), proline (61.2), and ammonium chloride (46). To obtain 200 mg/l of yeast assimilable nitrogen in the SM, 6.57 ml of this solution was added to the 1 l of medium.

Instead of adding ergosterol (yeast sterol) as described previously (Bely et al., 1990), SM medium was initially supplemented with anaerobic factors composed of phytosterols (85451, Sigma Aldrich), sterols naturally present in the grape juice (Le Fur et al., 1994), and Tween 80 for a final concentration of 10 mg/l. The stock solution was composed of 5 g/l of phytosterols in Tween 80 and ethanol (1:1, v/v).

The pH of the synthetic medium was adjusted to 3.3 with potassium hydroxide (Saarchem, Krugersdorp, South Africa). The trace elements, vitamins, nitrogen sources, and anaerobic factors were filtered through a 0.22-μm syringe filter (Starlab Scientific, Cape Town, South Africa) and added into the autoclaved synthetic medium.

Each fermentation was performed in triplicate. The fermentations were carried out in cylindrical fermenters of 3.5 cm diameter and 10 cm height. The fermenters contained 70 ml of medium, so that the headspace occupied 30% of the volume of the fermenters. In order to maintain anoxic conditions, the fermenters were equipped with fermentation locks filled with water, at 25°C, with orbital agitation (125 rpm). The fermentation progress was monitored by determination of CO_2_ release extrapolated from the measurement of the weight loss throughout the process.

At the end of each fermentation, different samples were centrifuged at 4000 g for 5 min, after which the supernatants were filtered through a 0.22-μm syringe filter (Starlab Scientific, Cape Town, South Africa) and stored at −20°C for further chemical analysis.

### Additions of nitrogen sources

For some fermentations where *Z. meyerae* or *K. marxianus* were sequentially inoculated with *S. cerevisiae*, nitrogen sources (ammonium, mixture of amino acids, or FermaidO®, Lallemand SAS, Canada) were added at the same time as the inoculation of *S. cerevisiae* to reach the yeast assimilable nitrogen concentration of 200 mg/l.

### Monitoring of yeast population

During the first 48 h, the yeast cell populations were monitored by plating each day the appropriate dilutions onto YPD nutrient agar (Biolab-Merck, Modderfontein, South Africa). After the *S. cerevisiae*'s inoculation, the viability of yeasts was monitored throughout the fermentation by plating on a selective medium which was identified before inoculation. The three non-*Saccharomyces* yeasts were enumerated on YPD agar supplemented with 5 mg/l cycloheximide, which was the lowest concentration found to suppress *S. cerevisiae* growth while allowing that of the other species. *S. cerevisiae*'s population was determined by plating the appropriate dilutions on YPD agar plates and by subtracting the yeasts enumerated on YPD + cycloheximide plates. Plates were incubated at 30°C, generally for 2–3 days, until colonies were formed.

### Quantification of residual sugars and ammonium by enzymatic assays

For the residual glucose, fructose, and ammonium concentrations, 400 μl of filtered sample was enzymatically analyzed using the Arena 20XT (Thermo Fisher Scientific, Waltham, MA) which makes use of automated spectrophotometric readings to determine the concentrations of the various compounds. The different enzymatic assay kits utilized were the following: Enzytec™ Fluid D-Glucose (Id-No: 5140, R-BiopharmAG, Germany) for glucose, Enzytec™ Fluid D-Fructose (Id-No: 5120, R-BiopharmAG, Germany) for fructose, and Enzytec™ Fluid Ammonia (Id-No: 5390, R-BiopharmAG, Germany) for ammonium.

### Quantification of individual amino acids

Quantification of individual amino acids was performed by high performance liquid chromatography (HPLC), Agilent 1100 (Agilent Technologies, Waldbronn, Germany) using pre-column derivatization and fluorescence detection based upon a method previously described (Henderson and Brooks, 2010) with some modifications to the derivatization and injection. A Poroshell HPH-C18 column (4.6 × 150 mm, 2.7 μm particle size; Agilent Technologies) was used following derivatization of the amino acids. Derivatization was performed using three different reagents: iodoacetic acid (Sigma Aldrich) for cysteine, o-phthaldialdehyde (OPA, Sigma Aldrich) for primary amino acids, and fluorenylmethyloxycarbonyl chloride (Sigma Aldrich) for secondary amino acids. Internal standards, norvaline (Sigma Aldrich), and sarcosine (Sigma Aldrich) were spiked to each sample prior to derivatization. One milliliter of each filtered sample was analyzed.

### Analysis of major volatile compounds

The quantification of major volatiles (i.e., a selection of higher alcohols, acetate esters, fatty acids, fatty acid ethyl esters) was carried out by gas chromatography equipped with a flame ionization detector (GC-FID) using the Agilent GC System HP 6890 Series (Agilent, Little Falls, Wilmington, USA) as described previously (Louw et al., 2009) with minor modifications. Five milliliters of each of the filtered samples were used with 100 μl of 4-methyl-2-pentanol (internal standard). Diethyl ether (1 ml) was added to the mixture which was then placed in an ultrasonic bath for 5 min to extract the volatile compounds. Thereafter, the samples were centrifuged at 4000 g for 3 min. Sodium sulfate was added to remove any water from the non-polar layer. HP Chemstation software was used for data analysis.

### Statistical analysis

Statistical analyses were performed using the R software, version 3.2.3 (http://cran.r-project.org/). Each variable was then tested using a one-way analysis of variance (ANOVA) with the uptake concentration of each nitrogen source as a factor to describe the diversity between the different strains to detect a global effect at a *p*-value threshold of 0.05. For each parameter, normality of residual distributions and homogeneity of variance were studied using standard diagnostic graphics; no violation of the assumptions was detected. As the effect was significant at a *p*-value threshold of 0.05, all pairwise comparisons for agitation speed were tested using Tukey's honestly significant difference (HSD) test. The principal component analysis (PCA) was carried out with the FactoMineR package (Le et al., 2008).

## Results

This work aimed to compare the outcomes of fermentation (fermentation performances and aroma production) by three non-*Saccharomyces* strains during sequential inoculations with *S. cerevisiae* and highlighted the possible competition for nutrients.

### Fermentation kinetics and population dynamics

**Figure 1:**
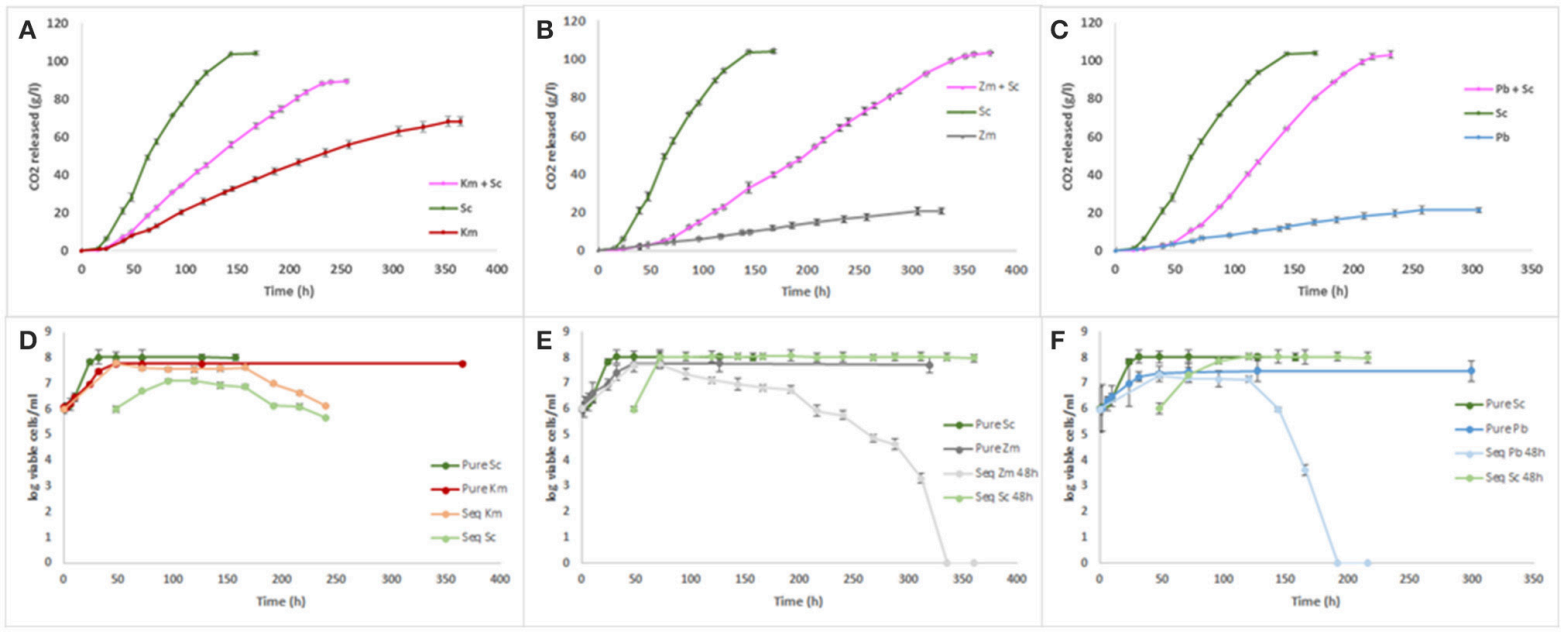
Fermentation kinetics and yeast growth of pure and sequential cultures of *K. marxianus* **(A,D)**, *Z. meyerae* **(B,E)**, and *P. burtonii* **(C,F)** with *S. cerevisiae*.

As expected, the pure culture of *S. cerevisiae*, considered as control, was the only yeast to reach dryness (i.e., residual sugars below 2 g/l), while fermentations conducted by the three non-*Saccharomyces* yeast pure cultures got stuck with residual sugars of 104, 184, and 190 g/l (data not shown) for *K. marxianus, P. burtonii*, and *Z. meyerae*, respectively (Figures 1A–C). Concerning the population dynamics, *S. cerevisiae* reached its maximal population (1.1 × 10^8^ cfu/ml) after 32 h of fermentation, while the three non-*Saccharomyces* species reached theirs after 48 h (6.10 × 10^7^, 5.8 × 10^7^, and 3.0 × 10^7^ cfu/ml, for *K. marxianus, Z. meyerae*, and *P. burtonii*, respectively). No loss of viability was observed in pure culture (Figures 1D–F).

Concerning the sequential inoculations, three different behaviors were observed according to the yeasts inoculated with *S. cerevisiae*. Only the sequential inoculation of *K. marxianus* together with *S. cerevisiae* did not reach dryness and got stuck with 48 g/l of residual sugar (data not shown), while the sequential fermentations with *P. burtonii* or *Z. meyerae* exhausted all the sugars (Figures 1A–C). However, the kinetic profiles were different. Indeed, the fermentation with *Z. meyerae* displayed a longer fermentation duration (around 15 days) with a slow average fermentation rate even after the inoculation of *S. cerevisiae* (0.35 g CO_2_/l/h) (Figure 1B). However, the fermentation performed with *P. burtonii* finished in 10 days displaying after the inoculation of *S. cerevisiae* the same average fermentation rate (0.93 g CO_2_/l/h) as the *S. cerevisiae*'s pure culture (Figure 1C).

The population dynamics during sequential fermentation presented two distinct patterns. For sequential inoculations with *Z. meyerae* or *P. burtonii*, a mortality of these yeasts was observed within the hours following the inoculation of *S. cerevisiae* until they were no longer detectable, while *S. cerevisiae*'s population reached the same maximal population than its pure culture (Figures 1E,F). Concerning the sequential culture with *K. marxianus*, a very weak implantation of *S. cerevisiae* was observed associated with a decrease of the non-*Saccharomyces* yeast population (Figure 1D). When fermentation stopped, both species were undetectable.

### Consumption of nitrogen sources

Consumption of ammonium and amino acids was determined at 48 h and at the end of fermentation during the sequential inoculations (Table 1). After 48 h of fermentation, the nature and the quantity of nitrogen sources consumed differed for each strain. *S. cerevisiae* displayed the highest uptake for the majority of nitrogen sources (55% of the assimilable nitrogen) except for γ-aminobutyric acid (GABA) and arginine which were taken up in greater amounts by *Z. meyerae* and *P. burtonii* for GABA and by *Z. meyerae* for arginine. *K. marxianus* was able to consume almost 40% of the assimilable nitrogen present in the medium within the first 48 h and displayed the same preferences than *S. cerevisiae*, except for ammonium which was poorly consumed by *K. marxianus*. On the other hand, *P. burtonii* and *Z. meyerae* were able to consume around 20% of the nitrogen. Interestingly within the first 48 h, *Z. meyerae* did not consume threonine at all. At the end of the sequential fermentations and for all the yeasts, nitrogen was completely depleted (except for GABA and in a lesser extent glycine).

**Table 1:** Uptake of individual amino acids and ammonium (in mg/l) for the 4 strains after 48 h and at the end of fermentation during sequential inoculations for the non-*Saccharomyces* yeasts and during pure culture for *S. cerevisiae*.

	**Initial content**	**After 48 h of fermentation**				**End of fermentation**			
		***S. cerevisiae***	***K. marxianus***	***Z. meyerae***	***P. burtonii***	***S. cerevisiae***	***K. marxianus***	***Z. meyerae***	***P. burtonii***
NH4	104.89	60.94 ± 0.54^a^	24.07 ± 0.28^b^	27.41 ± 1.29^c^	22.93 ± 1.19^b^	104.89 ± 0.21^a^	104.88 ± 0.32^a^	104.92 ± 0.43^a^	104.86 ± 0.54^a^
GLU	80.30	46.39 ± 0.15^a^	28.12 ± 1.28^b^	5.47 ± 0.67^c^	14.70 ± 1.72^d^	80.31 ± 0.02^a^	80.29 ± 1.83^a^	78.81 ± 0.74^b^	80.30 ± 1.29^a^
GLN	303.34	205.53 ± 0.07^a^	156.55 ± 6.56^b^	52.86 ± 1.50^c^	95.73 ± 1.80^d^	303.34 ± 10.23^a^	298.92 ± 10.76^a^	303.34 ± 18.96^a^	303.34 ± 4.87^a^
ARG	229.10	77.89 ± 0.43^a^	49.06 ± 4.22^b^	99.10 ± 0.15^c^	35.60 ± 0.12^d^	229.09 ± 11.23^a^	223.64 ± 12.50^a^	228.16 ± 19.50^a^	229.10 ± 20.37^a^
ASP	28.40	19.67 ± 0.19^a^	13.48 ± 0.16^b^	3.99 ± 0.30^c^	9.03 ± 0.09^d^	28.40 ± 0.11^a^	28.39 ± 0.79^a^	28.32 ± 3.06^a^	28.40 ± 0.52^a^
ASN	41.14	19.50 ± 0.03^a^	18.12 ± 0.45^b^	6.42 ± 0.16^c^	11.19 ± 0.52^d^	41.14 ± 1.21^a^	39.22 ± 2.24^a^	40.24 ± 4.44^a^	41.14 ± 1.83^a^
HIS	24.56	23.86 ± 0.06^a^	10.21 ± 0.34^b^	1.28 ± 0.56^c^	4.99 ± 0.23^d^	24.56 ± 0.32^a^	24.56 ± 0.78^a^	24.56 ± 3.58^a^	24.56 ± 0.03^a^
GLY	16.06	8.01 ± 0.06^a^	0.85 ± 0.40^b^	1.51 ± 0.08^c^	4.18 ± 0.26^d^	16.06 ± 0.35^a^	13.52 ± 2.31^b^	14.01 ± 3.19^b^	16.06 ± 0.93^a^
ALA	92.14	36.89 ± 0.26^a^	24.88 ± 2.52^b^	16.13 ± 0.78^c^	14.84 ± 1.42^c^	92.14 ± 5.03^a^	90.46 ± 4.94^a^	90.48 ± 8.78^a^	92.14 ± 4.98^a^
GABA	142.82	12.18 ± 1.01^a^	17.92 ± 1.87^b^	47.26 ± 0.69^c^	19.07 ± 2.45^d^	34.09 ± 7.43^a^	37.99 ± 9.87^a^	88.93 ± 11.85^b^	41.96 ± 7.86^c^
LYS	12.11	11.24 ± 0.13^a^	10.93 ± 0.11^c^	4.05 ± 0.10^b^	11.39 ± 0.21^c^	12.11 ± 0.97^a^	12.11 ± 0.31^a^	12.11 ± 1.62^a^	12.11 ± 0.39^a^
SER	56.01	43.85 ± 0.09^a^	28.89 ± 0.65^b^	7.64 ± 0.25^c^	16.98 ± 0.52^d^	56.11 ± 0.02^a^	56.09 ± 0.21^a^	56.01 ± 0.96^a^	56.07 ± 0.24^a^
THR	45.53	36.57 ± 0.05^a^	23.63 ± 0.84^b^	0.02 ± 0.24^c^	15.32 ± 0.04^d^	45.59 ± 0.19^a^	45.53 ± 0.81^a^	45.51 ± 0.64^a^	45.56 ± 0.16^a^
TYR	13.94	11.40 ± 0.06^a^	7.36 ± 0.06^b^	2.48 ± 0.12^c^	2.97 ± 0.26^d^	13.99 ± 0.34^a^	13.94 ± 0.22^a^	13.91 ± 0.23^a^	13.92 ± 0.84^a^
VAL	34.32	28.27 ± 0.26^a^	25.76 ± 0.14^b^	5.78 ± 0.09^c^	7.52 ± 0.60^d^	34.33 ± 0.18^a^	34.32 ± 0.25^a^	34.30 ± 0.15^a^	34.30 ± 0.58^a^
MET	16.88	17.12 ± 0.32^a^	14.81 ± 0.21^b^	8.61 ± 0.27^c^	3.92 ± 0.16^d^	16.90 ± 0.17^a^	16.88 ± 0.12^a^	16.88 ± 0.67^a^	16.88 ± 0.03^a^
TRP	118.46	75.17 ± 0.07^a^	43.61 ± 1.88^b^	27.31 ± 0.28^c^	31.96 ± 0.11^d^	118.46 ± 1.43^a^	116.48 ± 1.57^a^	118.46 ± 1.06^a^	118.46 ± 2.93^a^
PHE	30.69	29.66 ± 0.06^a^	27.55 ± 0.25^b^	5.66 ± 0.25^c^	3.17 ± 0.67^d^	30.71 ± 0.76^a^	30.69 ± 0.10^a^	30.68 ± 0.90^a^	30.70 ± 0.41^a^
ILE	20.16	19.50 ± 0.08^a^	19.22 ± 0.06^a^	3.95 ± 0.15^b^	7.94 ± 0.31^c^	20.16 ± 0.32^a^	20.18 ± 0.06^a^	20.12 ± 0.75^a^	20.13 ± 0.24^a^
LEU	32.66	32.79 ± 0.02^a^	31.64 ± 0.08^b^	4.78 ± 0.22^c^	15.33 ± 0.37^d^	32.66 ± 1.34^a^	32.61 ± 0.25^a^	32.63 ± 0.42^a^	32.68 ± 0.30^a^
Total	1443.5^*^	816.43	576.66	331.71	348.76	1335.04	1320.7	1382.38	1342.67

*Mean values ± standard deviation Strains sharing the same letter for a nitrogen sources are not significantly different at a 0.05 threshold*.

* *1443.5 mg/l corresponds to 200 mg/l of assimilable nitrogen. Proline was not consumed by the different yeast strains (data not shown). Due to the HPLC method, cysteine concentration was not correctly assessed*.

### Production of major volatile compounds during sequential fermentations

**Figure 2:**
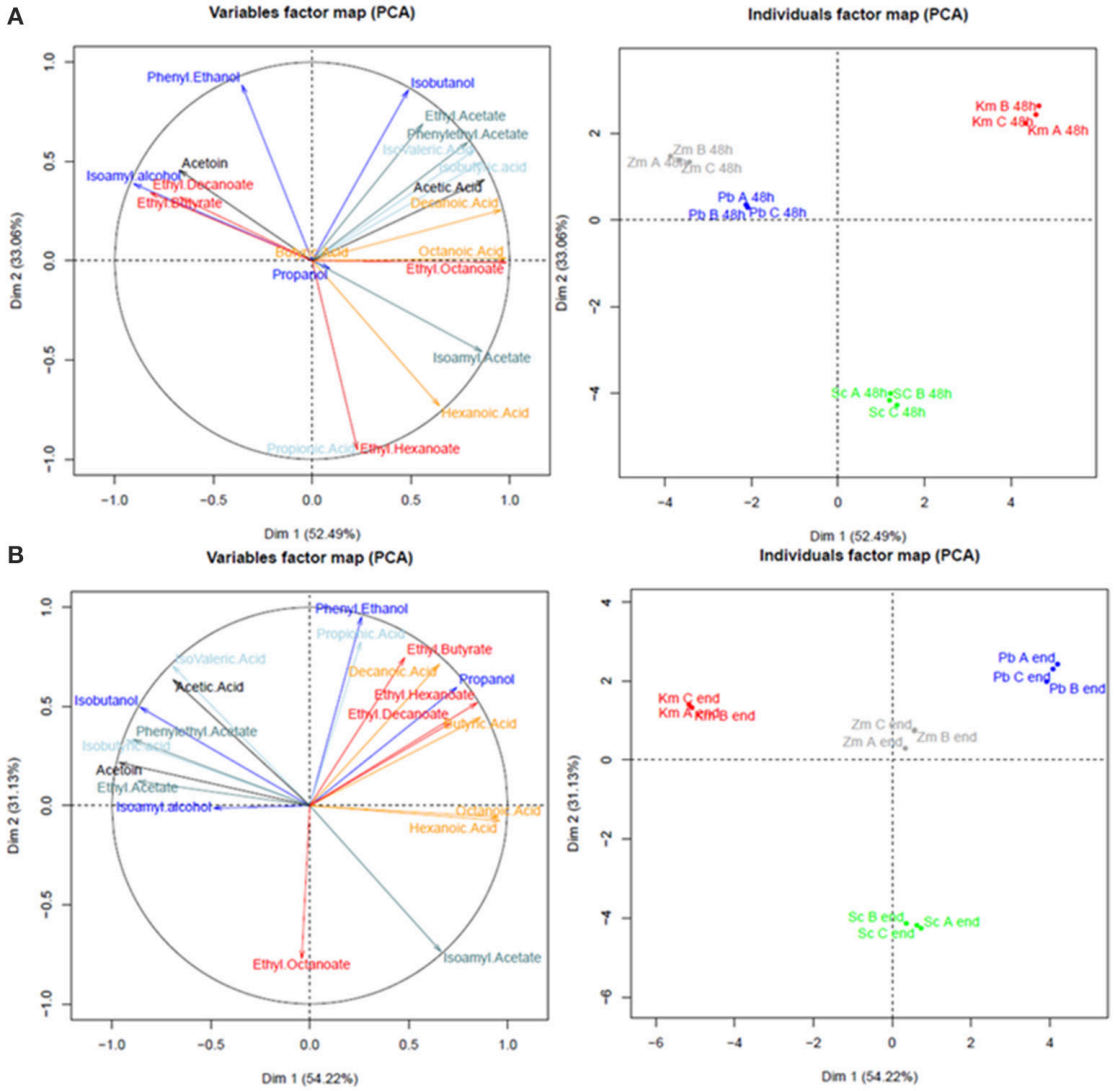
Principal component analysis of major volatile compound production after 48 h **(A)** and at the end of fermentation **(B)** during sequential fermentations. Dark blue, higher alcohols; light blue, acetate esters; gray blue, fusel acids; red, small and medium chain fatty acids; orange, ethyl esters.

The major volatile compounds formed by yeasts during alcoholic fermentation were determined after 48 h of fermentation and in the final wines (Figure 2A,B, Table SD1). First, it is interesting to note that the triplicates were well grouped on the PCA and three groupings were identifiable: *Z. meyerae* and *P. burtonii, K. marxianus* and finally *S. cerevisiae*. After 48 h of fermentation, different aroma profiles can be identified according to the yeast species inoculated. Fermentations performed with *K. marxianus* were characterized by the enhanced production of isobutanol, phenylethylacetate, ethyl acetate, and acids (fusel and medium chain fatty acids), while *Z. meyerae* and *P. burtonii* produced the highest amount of phenylethanol, isoamyl alcohol, and ethyl esters (Figure 2A). Isoamyl acetate, ethyl hexanoate, hexanoic, and propionic acids characterized the fermentations conducted by *S. cerevisiae* alone (Figure 2A). Interestingly, *Z. meyerae* and *P. burtonii* did not produce any fatty acid (short or medium chain) within the first 48 h, but at the end of the sequential fermentation, the concentrations were higher than the pure culture of *S. cerevisiae* (with just a few exceptions). In the final wines, the groupings remained the same than after 48 h, and were still characterized by the same aromatic profiles according the non-*Saccharomyces* used to perform the sequential inoculation with *S. cerevisiae* with some notable exceptions such as isoamyl alcohol, acetoin, and some fatty acids (Figure 2B).

**Figure 3:**
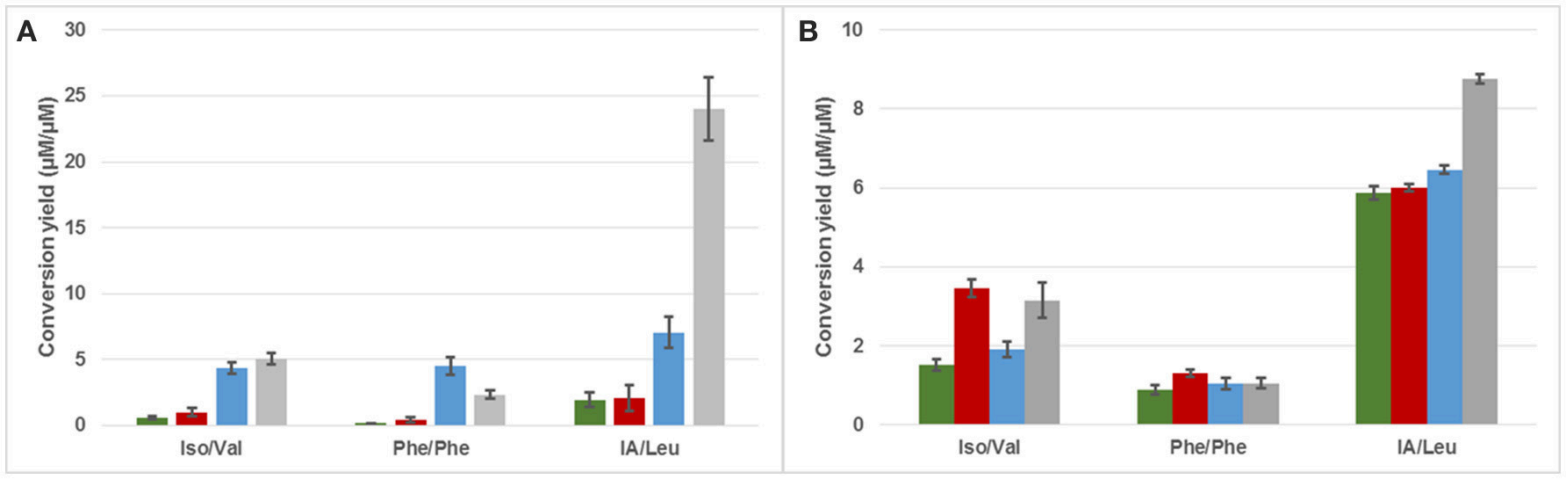
Conversion yield of amino acid precursors into their higher alcohols at 48 h **(A)** and at the end of fermentation **(B)**. ISO, isobutanol; Phe, phenylethanol; IA, isoamyl alcohol; Val, valine; Phe, phenylalanine; Leu, leucine. Green: *S. cerevisiae*, Red: *K. marxianus*, Blue: *P. burtonii*, Gray: *Z. meyerae*.

Higher alcohols can be formed by the catabolism of certain amino acids (via the Ehrlich pathway) but also by the sugar metabolism. In an attempt to estimate the amount of these compounds which was directly formed through amino acid metabolism (in contrast to that formed through carbon metabolism), the molar ratio of higher alcohol produced over the amino acid precursor consumed was calculated (Figure 3). At 48 h, this ratio was systematically higher for *Z. meyerae* and *P. burtonii* (e.g., 12 and 4 times higher for isoamyl alcohol/leucine, respectively) than that calculated for the pure culture of *S. cerevisiae*, while *K. marxianus* and *S. cerevisiae* displayed the same ratio (Figure 3A). At the end of fermentation, the differences between the yeast strains were less important, but some differences were still visible. *S. cerevisiae* presented the lowest ratio for the three higher alcohols (Figure 3B). The ratios for isobutanol and phenylethanol were higher for the wines obtained with *K. marxianus* (2 and 1.5 times higher respectively, Figure 3B) than those obtained for *S. cerevisiae*'s pure culture. Concerning isoamyl alcohol, the highest ratio was reached with *Z. meyerae* and then *P. burtonii* (1.5 and 1.2 time higher, respectively). The same trends were observed for the fusel acids and acetate esters (data not shown).

**Figure 4:**
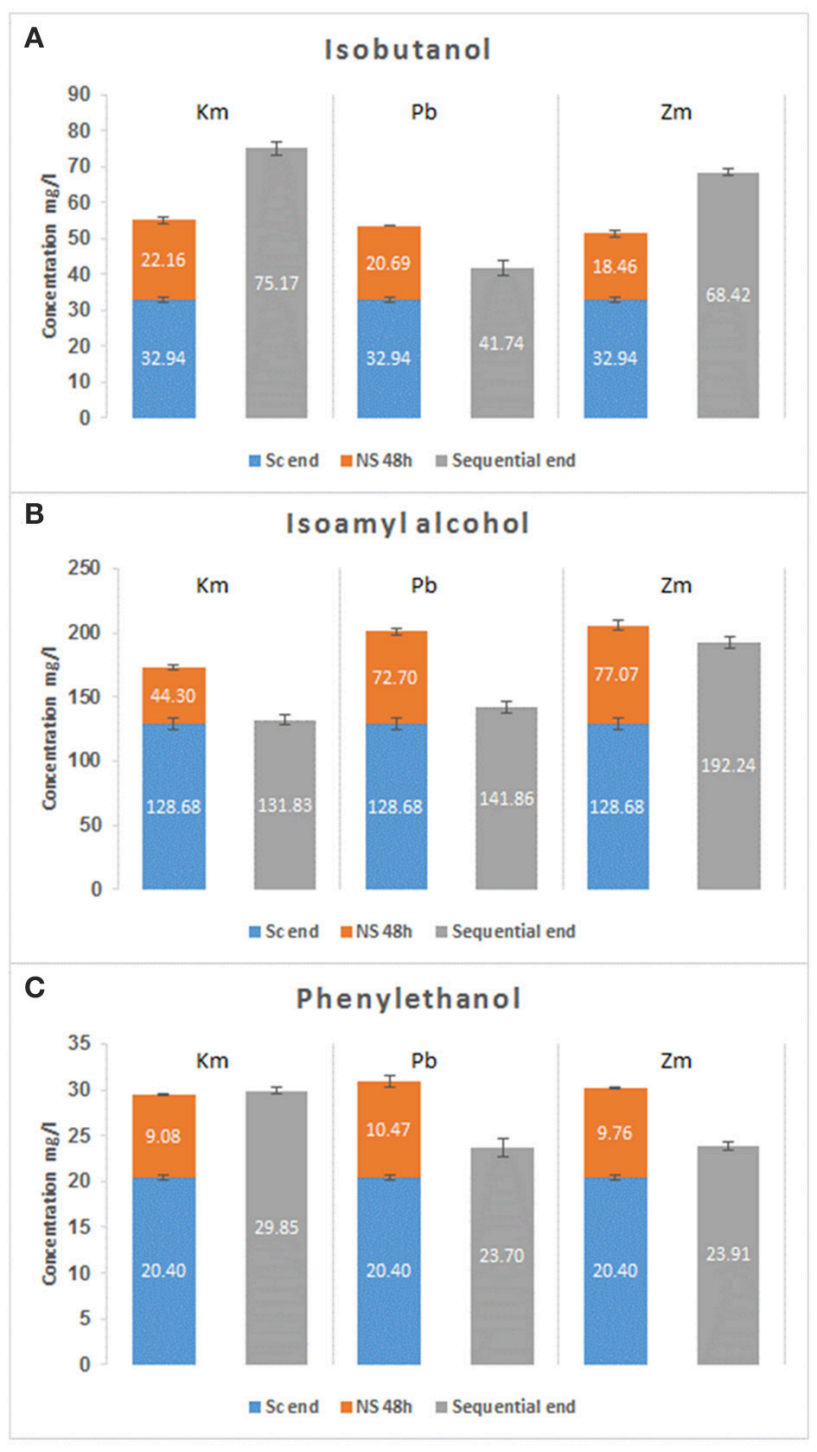
Contribution of non-Saccharomyces yeasts for isobutanol **(A)**, isoamyl alcohol **(B)**, and phenylethanol **(C)** production during sequential fermentations.

At the end of the fermentation, the concentrations of isobutanol, phenylethanol, and isoamyl alcohol were generally much higher in sequential fermentations than in those where non-*Saccharomyces* yeasts were absent (Figure 4). The figure shows that the contribution of non-*Saccharomyces* yeasts extends beyond the concentrations produced during the first 48 h, such as the production of isobutanol with *K. marxianus* or *Z. meyerae*.

### Influence of additions of various nitrogen sources

**Figure 5:**
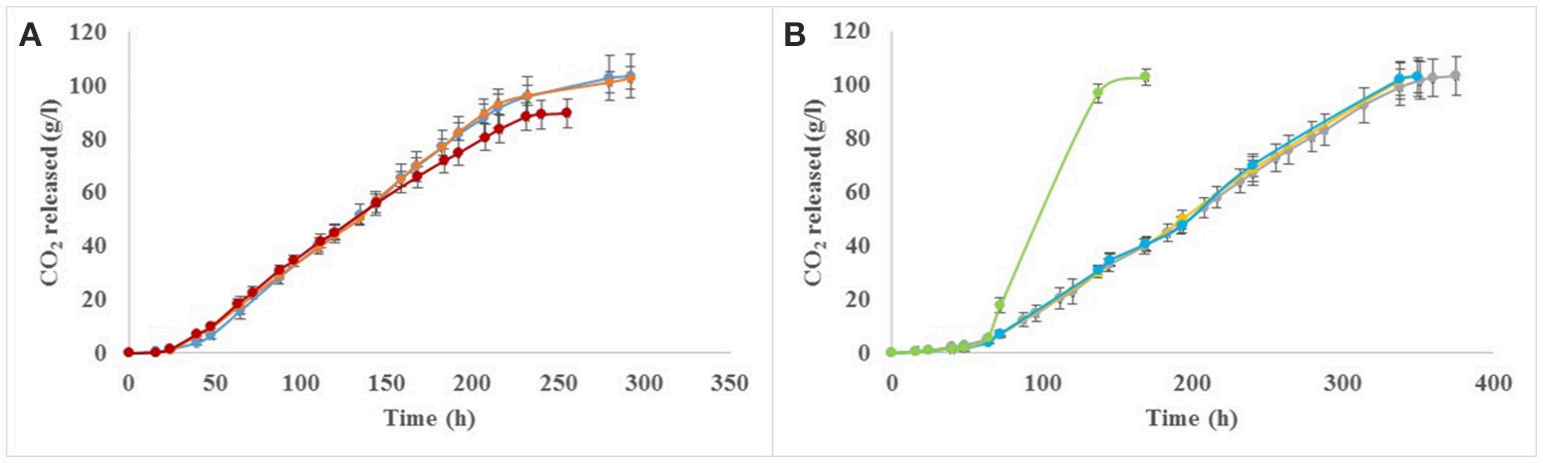
Impact of nitrogen additions on fermentation performances of *K. marxianus* **(A)** and *Z. meyerae* **(B)**. Ammonium addition (orange), amino acid mixture addition (blue), FermaidO® addition (green), no addition with *K. marxianus* (red), or *Z. meyerae* (gray).

As *K. marxianus* consumed 70% of the assimilable nitrogen available during the first 48 h of fermentation and considering the fact that *S. cerevisiae* was not able to grow, nitrogen additions (with ammonium or mixture of amino acids and ammonium used in the synthetic must) were performed (Figure 5A). With nitrogen supplementations, the fermentation performed with *K. marxianus* sequentially inoculated with *S. cerevisiae* was able to reach dryness regardless of the nature of the nitrogen source added (Figure 5A).

When *Z. meyerae* was sequentially inoculated with *S. cerevisiae*, the fermentation reached dryness but only after 15 days due to a slower fermentation rate compared to the *S. cerevisiae*'s pure culture or the sequential inoculation *P. burtonii*/*S. cerevisiae* (Figure 1). To determine the reason of this slower fermentation, some nutrient additions were performed: mixture of amino acids, ammonium or a commercial nutrient (yeast autolysate), FermaidO®. No impact on the fermentation process was observed with the addition of amino acids and ammonium, while the fermentation was shorter with the addition of the commercial nutrient (Figure 5B). The limitation of the fermentation performance was not due to nitrogen deficiency.

## Discussion

The inoculation of non-*Saccharomyces* yeasts sequentially with *S. cerevisiae* is becoming a common practice to alter the organoleptic properties of wine (Hu et al., 2016; Liu et al., 2016; Lleixà et al., 2016; Renault et al., 2016; Gobert et al., 2017). However, the yeast-yeast interactions and possible competition for nutrients arising from this new style of inoculation require further investigations (Ciani and Comitini, 2011; García et al., 2016). Previously, it was suggested that the growth of non-*Saccharomyces* yeasts resulted in the depletion in nutrients, especially in assimilable nitrogen, and in an unfavorable medium for *S. cerevisiae* proliferation (Fleet, 1993; Bataillon et al., 1996). The aim of our study was to evaluate the effect of sequential *S. cerevisiae* and non-*Saccharomyces* cultures on nitrogen source consumption and fermentative aroma production in order to determine the extent of competition for nitrogen sources among the different microorganisms involved and ultimately the consequence on fermentation outcomes.

Our results clearly demonstrate that *S. cerevisiae* had an antagonistic impact upon *P. burtonii* and *Z. meyerae* as the populations of these two species were rapidly decimated after the inoculation of *S. cerevisiae*. According to literature, several mechanisms underlying these interactions occur and depend on the *S. cerevisiae*/non-*Saccharomyces* pair used. Previous studies hypothesized that the premature death of non-*Saccharomyces* yeasts was induced by the production of toxic compounds by *S. cerevisiae* such as killer toxins or antimicrobial peptides (Pérez-Nevado et al., 2006; Albergaria and Arneborg, 2016; Wang et al., 2016). Other authors concluded that the early death of non-*Saccharomyces* yeasts can be due to a cell-to-cell contact mechanism (Nissen et al., 2003; Renault et al., 2013; Kemsawasd et al., 2015a). It is important to note that in the present work, non-*Saccharomyces* yeasts and *S. cerevisiae* were inoculated at the same concentration (10^6^ cells/ml). Several studies highlighted that the increase of the ratio of inoculation in favor of the non-*Saccharomyces* yeasts improved their persistence in the medium in presence of *S. cerevisiae* (Pérez-Nevado et al., 2006; Comitini et al., 2011; Domizio et al., 2011). Changing this ratio could be a lever to enhance the persistence of these non-*Saccharomyces* yeasts in our fermentations but it would also increase the possible competition for nutrients without the guarantee of a better fermentation performance of non-*Saccharomyces* yeasts which displayed a very low sugar consumption in pure culture (<40 g/l).

The decline and premature death of *K. marxianus* during the sequential fermentation could be explained by its difficulty to overcome the ethanol increase and the impact on its plasmic membrane. Indeed, Diniz et al. (2017) demonstrated that the expression of some gene-encoding enzymes related to unsaturated fatty acid and ergosterol biosynthesis decreased upon ethanol exposure, and free fatty acid and ergosterol measurements demonstrate that their content in *K. marxianus* did not change under this stress.

Competition for nutrients may have a negative impact on *S. cerevisiae*'s growth and fermentation performance. Indeed, the uptake of nutrients by non-*Saccharomyces* yeasts may hinder *S. cerevisiae*'s growth and ultimately affect its fermentation performance. Non-*Saccharomyces* species growing early in the fermentation could strip the medium of amino acids and vitamins, limiting the subsequent growth and fermentation performances of *S. cerevisiae* (Bisson, 1999; Medina et al., 2012; Taillandier et al., 2014; Barbosa et al., 2015). In our study, three different behaviors of *S. cerevisiae* were observed depending on the non-*Saccharomyces* yeasts used in the sequential culture and can be explained by the competitions for nutrients. These species-dependent interactions were not observed by Gobert et al. (2017). Indeed, in the latter author' study, the performances of *S. cerevisiae* remained similar, regardless of the non-*Saccharomyces* inoculated. These differences in the behavior of *S. cerevisiae* could be explained by the differing experimental conditions between the two studies: (1) the species and the medium used were different, and (2) in this study, prior to inoculation into the fermentation medium, the yeasts were starved of nitrogen which probably greatly impacted their nitrogen uptake. During the sequential fermentation with *P. burtonii, S. cerevisiae* displayed the same performances than its pure culture and it can be concluded that no nutrient competition occurred between these two strains, corroborated by the very low amino acid and ammonium consumption of *P. burtonii*. While *S. cerevisiae* grew poorly and declined rapidly during the sequential culture with *K. marxianus* leading to an incomplete fermentation. The competition for nitrogen between *K. marxianus* and *S. cerevisiae* was suspected because of the amino acid consumption pattern of *K. marxianus* was very close to that of *S. cerevisiae* and then confirmed experimentally when ammonium or amino acid additions led to a complete fermentation. This observation was consistent with previous studies conducted on other species which demonstrated this competition between *T. delbrueckii* (Taillandier et al., 2014) or *L. thermotolerans* (Ciani et al., 2006; Gobbi et al., 2013) and *S. cerevisiae*. Concerning the sequential culture *Z. meyerae*/*S. cerevisiae*, competition for another nutrient different from nitrogen was evidenced. The addition of amino acids or ammonium did not change the fermentation rate of *S. cerevisiae*. However, the addition of a more complex nutrient led to a faster fermentation, thereby suggesting that the strains competed for lipids, vitamins or minerals. Indeed, literature in reference to the vitamin requirements for growth and fermentation performances by wine yeasts is very limited. Bataillon et al. (1996) showed that *K. apiculata* was very efficient at shipping thiamine and removed this vitamin from the medium more rapidly than *S. cerevisiae* leading to deficient growth of *S. cerevisiae*. Recently, Medina et al. (2012) also highlighted the importance of vitamin availability during mixed cultures. Moreover, the use of complex nutrients can also be an alternative to restore a certain balance between the various nutrients, especially by providing lipids. Indeed, it was previously shown that a deficiency in lipids leads to stuck or sluggish fermentations and the addition of lipids allows to re-establish a complete fermentation, a better growth and viability of cells (Casalta et al., 2013; Ochando et al., 2017).

Most of the studies with co-inoculation or sequential inoculation of non-*Saccharomyces*/*S. cerevisiae* species have highlighted the differences in the aromatic profiles obtained in these final wines compared with monocultures of *S. cerevisiae* (Comitini et al., 2011; Andorrà et al., 2012; Renault et al., 2015; Gobert et al., 2017). However, none of the latter studies clearly established that the aroma compounds produced by the non-*Saccharomyces* yeasts within the first 48 h (prior to *S. cerevisiae* inoculation) allow to distinguish the final wines from each other in a species-dependent manner. In this context, our study clearly demonstrated that the aromatic footprint of the non-*Saccharomyces* yeasts visible after 48 h was still present at the end of the sequential culture, irrespective of the survival or decline of these yeasts. For instance, *P. burtonii* and *Z. meyerae* were associated with a higher production of higher alcohols. These compounds can have both a positive and negative impact on the aroma and flavor of a wine depending on their final concentration (Beltran et al., 2005). It has been reported that concentrations below 300 mg/l add a desirable level of complexity to wine, whereas concentrations that exceed 400 mg/l can have a detrimental effect. Both the sequential cultures *P. burtonii*/*S. cerevisiae* and *Z. meyerae*/*S. cerevisiae* never exceeded this concentration (Table SD1). These strains also significantly increased the synthesis of ethyl esters that impart fruity flavors to wine associated with the increase of short and medium chain fatty acids, precursors of these esters. The sequential fermentation with *K. marxianus* presented significant increases in compounds, which can impact positively on the aroma such as phenylethanol and phenylethyl acetate, which contribute to a desirable floral (rose) aroma, consistent with previous observations about this species (Gethins et al., 2015). The final result of these fermentations will be a higher complexity, yet further studies including sensorial analysis should be performed. Nevertheless, we cannot certify the origin of this higher complexity. The different aromatic patterns of wines can be due to: (i) the production of volatile compounds throughout the fermentation by the non-*Saccharomyces* yeasts even after the inoculation of *S. cerevisiae*, or (ii) the interaction between non-*Saccharomyces* yeast and *S. cerevisiae* which impacted the metabolism of the latter which will then produce more aromas than in pure culture. Gobert et al. (2017) also suggested the existence of these two mechanisms which appeared to be volatile compound- and strain-dependent in their study.

Higher alcohols can be formed from the degradation of specific amino acids or from sugar metabolism (Hazelwood et al., 2008). Previous studies on *S. cerevisiae* showed that only a small fraction (5%) of higher alcohols were produced from the catabolism of amino acids (Crépin et al., 2017; Rollero et al., 2017). Our results suggested that this fraction was even smaller for *Z. meyerae* and *P. burtonii* because of their very low consumption of valine, leucine and phenylalanine and comparatively high production of the corresponding higher alcohols (Table 1), while it remained similar for *K. marxianus*, except for the phenylalanine/phenylethanol ratio which seemed to be higher. Since phenylethanol may be produced through the degradation of compounds arising from the pentose phosphate pathway, this observation is in accordance with the transcriptomic results reported in Diniz et al. (2017). Indeed, the latter authors showed that the genes involved in the pentose phosphate pathway seemed to be overexpressed in the presence of 6% of ethanol. A complete quantitative study of the fate of amino acids is required to better characterize the role of amino acids in the aroma production.

## Conclusion

The use of sequential yeast cultures in industrial wine production is currently under scrutiny. In this study, we demonstrated that the nitrogen consumption by non-*Saccharomyces* yeasts during sequential fermentations with *S. cerevisiae* can lead to stuck or sluggish fermentation due to species-dependent competition for nitrogen sources but also for other nutrients, thereby highlighting the importance of monitoring nutrient concentrations closely in these inoculation scenarios. Nevertheless, our study also showed that the use of non-*Saccharomyces* yeasts led to a more complex and aromatic wine that the monoculture of *S. cerevisiae*. These benefits could justify the selection of appropriate non-*Saccharomyces* yeasts whose production of detrimental products is low and that they interact correctly with *S. cerevisiae*. Thus, a better understanding of the nutrient consumption is required for industrial environments in order to adapt nitrogen management according to the yeast pair considered.

## Author contributions

SR: Performing the experiments, writing of the manuscript, discussion of results, analysis of results. AB, CC, and BD: Design of experiments, analysis of results, discussion of results, writing of the manuscript. AO-J: Design of experiments, discussion of the results.

### Conflict of interest statement

The authors declare that the research was conducted in the absence of any commercial or financial relationships that could be construed as a potential conflict of interest.
