# Supplementary material for: Altered Fermentation Performances, Growth, and Metabolic Footprints Reveal Competition for Nutrients between Yeast Species Inoculated in Synthetic Grape Juice-Like Medium
Source: Front Microbiol. 2018 Feb 13;9:196. doi: 10.3389/fmicb.2018.00196 (PMC5816954; doi:10.3389/fmicb.2018.00196)
Supplement: Supplementary file 1 [file Table1.DOCX]

Supplementary Material

Altered fermentation performances and metabolic footprints reveal competition for nutrients between yeast species inoculated in synthetic grape juice-like medium

Stephanie Rollero, Audrey Bloem, Anne Ortiz-Julien, Carole Camarasa, Benoit Divol^*^

*** Correspondence:** Benoit Divol: [divol@sun.ac.za](mailto:divol@sun.ac.za)

**Table SD1:** Volatile compound concentration (in mg/l) during sequential fermentations

|  | **After 48h of fermentation** | | | | **End of fermentation** | | | | |  |
| --- | --- | --- | --- | --- | --- | --- | --- | --- | --- | --- |
|  | ***S. cerevisiae*** | ***K. marxianus*** | ***Z. meyerae*** | ***P. burtonii*** | | ***S. cerevisiae*** | ***K. marxianus*** | ***Z. meyerae*** | ***P. burtonii*** | |
| **Propanol** | 13.89 ± 0.05 | 15.41 ± 0.21 | 6.36 ± 0.06 | 26.36 ± 0.18 | | 14.69 ± 0.15 | 16.29 ± 0.25 | 13.22 ± 0.25 | 36.25 ± 0.29 | |
| **Isobutanol** | 10.49 ± 0.13 | 22.16 ± 1.03 | 18.46 ± 0.12 | 20.69 ± 0.77 | | 32.94 ± 0.51 | 75.17 ± 1.75 | 68.42 ± 0.98 | 41.74 ± 2.13 | |
| **Isoamyl alcohol** | 42.55 ± 2.23 | 44.30 ± 2.24 | 77.07 ± 3.68 | 72.70 ± 2.54 | | 128.68 ± 4.65 | 131.83 ± 3.96 | 192.24 ± 4.98 | 141.86 ± 4.63 | |
| **Phenylethanol** | 3.29 ± 0.08 | 9.08 ± 0.14 | 9.76 ± 0.07 | 10.47 ± 0.63 | | 20.40 ± 0.22 | 29.85 ± 0.38 | 23.91 ± 0.44 | 23.70 ± 1.03 | |
| **Ethyl Acetate** | 48.9 ± 2.31 | 59.01 ± 0.38 | 31.61 ± 0.35 | 3.37 ± 0.02 | | 62.02 ± 4.73 | 79.36 ± 1.46 | 74.38 ± 1.36 | 53.68 ± 1.05 | |
| **Isoamyl acetate** | 0.28 ± 0.01 | 0.23 ± 0.01 | nd | nd | | 1.27 ± 0.02 | 0.60 ± 0.03 | 0.96 ± 0.03 | 0.99 ± 0.02 | |
| **Phenylethyl acetate** | 0.49 ± 0.02 | 2.62 ± 0.46 | 0.67 ± 0.03 | 0.47 ± 0.01 | | 1.44 ± 0.07 | 4.69 ± 0.24 | 1.49 ± 0.05 | 1.50 ± 0.12 | |
| **Propionic acid** | 0.15 ± 0.01 | nd | nd | nd | | 0.66 ± 0.03 | 2.30 ± 0.07 | 3.90 ± 0.43 | 3.38 ± 0.35 | |
| **Isobutyric acid** | 0.29 ± 0.01 | 3.83 ± 0.32 | nd | nd | | 1.28 ± 0.03 | 5.40 ± 0.14 | 1.89 ± 0.30 | 1.18 ± 0.27 | |
| **Isovaleric acid** | nd | 1.12 ± 0.02 | nd | nd | | 0.27 ± 0.01 | 1.82 ± 0.03 | 1.15 ± 0.18 | 0.85 ± 0.04 | |
| **Butyric acid** | nd | nd | nd | nd | | 0.32 ± 0.06 | nd | 0.62 ± 0.19 | 1.73 ± 0.36 | |
| **Hexanoic acid** | 0.41 ± 0.01 | 0.19 ± 0.02 | nd | nd | | 1.48 ± 0.03 | 0.32 ± 0.04 | 1.38 ± 0.08 | 2.02 ± 0.15 | |
| **Octanoic acid** | 0.22 ± 0.02 | 0.38 ± 0.08 | nd | nd | | 1.44 ± 0.13 | 0.51 ± 0.03 | 1.36 ± 0.16 | 1.86 ± 0.09 | |
| **Decanoic acid** | 0.18 ± 0.01 | 0.55 ± 0.09 | nd | nd | | 0.60 ± 0.02 | 0.8 ± 0.04 | 1.16 ± 0.09 | 1.60 ± 0.24 | |
| **Ethyl butyrate** | nd | nd | 0.31 ± 0.01 | nd | | 0.44 ± 0.01 | 0.57 ± 0.02 | 0.83 ± 0.05 | 0.80 ± 0.04 | |
| **Ethyl hexanoate** | 0.11 ± 0.01 | nd | nd | nd | | 0.64 ± 0.01 | 0.60 ± 0.01 | 0.77 ± 0.04 | 0.92 ± 0.01 | |
| **Ethyl octanoate** | 0.17 ± 0.01 | 0.28 ± 0.01 | nd | nd | | 1.26 ± 0.01 | 0.55 ± 0.05 | 1.17 ± 0.06 | 0.30 ± 0.09 | |
| **Ethyl decanoate** | 0.22 ± 0.01 | 0.21 ± 0.01 | 0.70 ± 0.02 | nd | | 0.75 ± 0.04 | 0.63 ± 0.06 | 1.33 ± 0.03 | 1.23 ± 0.05 | |
| **Acetoin** | 7.38 ± 0.37 | 8.76 ± 0.88 | 16.64 ± 1.43 | 9.00 ± 0.42 | | 24.51 ± 0.26 | 41.17 ± 2.13 | 28.89 ± 1.59 | 20.59 ± 2.18 | |
| **Acetic acid** | 250.54 ± 16.75 | 518.11 ± 29.00 | 161.22 ± 33.81 | 289.62 ± 25.34 | | 608.49 ± 25.63 | 1036.60 ± 22.78 | 739.67 ± 19.99 | 856.66 ± 21.52 | |

Mean of three biological replicates ± standard deviation
